# The retinoic acid-producing capacity of gut dendritic cells and macrophages is reduced during persistent *T. muris* infection

**DOI:** 10.1111/pim.12032

**Published:** 2013-07-01

**Authors:** R J M Hurst, K J Else

**Affiliations:** The University of ManchesterManchester, UK

**Keywords:** aldehyde dehydrogenase, dendritic cells, macrophages, retinoic acid, *Trichuris muris*

## Abstract

Trichuris muris is an intestinal nematode that invades the colonic epithelium triggering a mucosal inflammation. Vitamin A and its active metabolite retinoic acid are strongly linked with the modulation of gut immune responses. Here, we describe the temporal changes in the expression of aldehyde dehydrogenase (ALDH) enzymes, responsible for converting dietary-absorbed vitamin A into the immuno-modulatory retinoic acid in lamina propria leucocytes post-infection. We show that ALDH enzymes are expressed by both colonic macrophages and dendritic cells. Further, during an on-going T. muris infection, ALDH expression is repressed from uninfected levels and only recovers to normal levels following expulsion of the parasite. These results suggest that local regulation of cellular levels of retinoic acid is an important component of infection-driven inflammation.

## Introduction

*Trichuris trichiura* is a nematode parasite that causes significant morbidity to more than 100 million people worldwide 1. *T. trichiura* is a colonic dwelling nematode, which buries into the epithelial lining to cause intestinal inflammation. In the laboratory, the mouse analogue of this parasite, *Trichuris muris*, is used to model and dissect the immune responses that occur during the human infection 2. Studies using *T. muris* have shown that the type of immune response that develops to the infection determines whether the host will be resistant or susceptible to this parasite. In short, a Th1 response makes a mouse susceptible to *T. muris* infection, whilst mouse strains that mount a Th2 response expel the parasite by approximately day 21 post-infection (p.i.) 3. Both the strain of mouse and the dose of *T. muris* eggs can determine which type of T helper cell response develops and hence the outcome of the infection. In this report, we utilize the susceptible AKR strain of mouse 4 and the mixed responding C57BL/6 strain. Giving a high dose *T. muris* infection of around 200 infective eggs to C57BL/6 mice results in an acute infection that is expelled from the host. In contrast, a low-dose infection of around 20–40 *T. muris* eggs results in a chronic infection 5.

Vitamin A has been linked with intestinal parasitic infections for many years 6. Deficiency in vitamin A colocalizes geographically with nematode infections 7, and studies have shown that re-infection of children in Mexico with *Ascaris lumbricoides* is reduced with vitamin A supplementation 8. Retinoic acid, the active metabolite of vitamin A, has been shown to be a key mediator of immune responses in the intestinal mucosa. As well as being important in the generation of regulatory T cells 9, it also plays roles in controlling the Th1/Th2 balance 10 and the expression of gut-homing receptors 11. Therefore, it is entirely possible that changes in vitamin A levels may have an impact on the outcome of intestinal parasite infections and/or the regulation of helminth-driven pathology. Given that people harbouring parasitic worms are often also vitamin A deficient and are therefore supplemented with this vitamin, it is critical to understand the relationship between vitamin A and intestinal nematodes.

Vitamin A (retinol) is obtained from the diet and converted into retinal by ubiquitously expressed alcohol dehydrogenases 12. Importantly, the final conversion from retinal to the active and highly immuno-modulatory metabolite retinoic acid is a tightly regulated process 13. It is controlled by three members of the aldehyde dehydrogenase (ALDH) family of enzymes, which are only expressed in some gut-associated immune cells 11, 14. ALDH1A1, ALDH1A2 and ALDH1A3, also known as the retinaldehyde dehydrogenases RALDH1, RALDH2 and RALDH3, respectively, have previously been shown to be limited to intestinal epithelial cells and MLN stromal cells, as well as, Peyer's patch, intestinal lamina propria and MLN dendritic cells (DCs) 9, 11, 15. This finely tuned control mechanism suggests a critical level for retinoic acid in directing local immune responses. Indeed, by quantifying RALDH2 expression by qPCR, previous studies have shown that retinoic acid production is decreased in the gut during a chemical model of colitis 16. Thus, understanding how the local production of retinoic acid changes during *T. muris* infection may help to determine whether retinoic acid is an important factor in controlling mucosal immunity during this parasite infection.

Here, we use the ALDEFLUOR assay to measure the expression of ALDH enzymes in gut-associated immune cells during acute and chronic models of *T. muris* infection. Although stromal cells and epithelial cells have been shown to express ALDH enzymes, we focus here on macrophages and DCs as key antigen-presenting cells important in instructing cells of the adaptive immune response. We show that both colonic macrophages and DCs express ALDH enzymes. We also find that the potential production of retinoic acid is decreased during a high-dose chronic infection, reflected in a significantly decreased percentage of ALDH^+^ macrophages and DCs. In the acute model of infection, however, the percentage of ALDH^+^ cells is restored following the expulsion of the parasite. Further, in a low-level chronic infection where only a few worms persist in the gut, the potential production of retinoic acid remains unchanged throughout the course of infection. Our results suggest that retinoic acid production is down-regulated during an inflammatory insult to the gut, and that a threshold level of inflammation exists, which is needed to drive the decrease in the percentage of ALDH^+^ macrophages and DCs in the gut.

## Materials and Methods

### Animals and parasites

Male AKR and C57BL/6 mice of 6–8 weeks of age were purchased from Harlan (UK) or Charles River (UK), respectively. All mice were housed at the BSF (University of Manchester) in sterile conditions, within individually ventilated cages. The Edinburgh (E) strain of *T. muris* was used and was maintained as previously described 17. AKR mice were infected with approximately 200 eggs, whilst C57BL/6 were infected with either approximately 200 eggs for an acute infection or 40–60 eggs to establish a chronic infection. All work was carried out in accordance with the UK Scientific Procedures (Animals) Act 1986.

Worm counts were not performed as gut tissue was used in lamina propria leucocyte isolation and flow cytometry. However, adult worms were visible in high-dose-infected AKR mice and low-dose-infected C57BL/6 mice at day 35 p.i., and absent from high-dose-infected C57BL/6 mice at day 35 and 42p.i., confirming their respective susceptibility and resistance to infection, as previously reported in the literature.

### Lamina propria leucocyte isolation and flow cytometry analysis

Lamina propria leucocytes (LPLs) were isolated from the large intestines of mice. The gut tissue was firstly washed in Hank's balanced salt solution (HBSS) containing 2% FCS (PAA Laboratories, GmbH, UK) and cut into segments. Epithelial cells were removed and discarded by shaking the tissue at 37°C in 2 mm EDTA in HBSS. The tissue was digested using a cocktail of enzymes comprised of collagenase V (Sigma, Poole, UK) (0·85 mg/ml), collagenase D (Roche, Welwyn Garden city, UK) (1·25 mg/ml), dispase (Gibco, Paisley, UK) (1 mg/ml) and DNase (Roche) (30μg/ml) at 37°C, and a single cell suspension was collected. Fc receptors were blocked using anti-CD16/CD32 antibodies (BD Biosciences, Oxford, UK). ALDH activity was assessed by flow cytometry using the ALDEFLUOR kit (Stem Cell Technologies, Manchester, UK) according to manufacturer's instructions. Cells were then stained with 7AAD (Cy5.5–PerCP channel) (BD Biosciences), AF700-conjugated anti-CD45 (BD Biosciences), efluor450-conjugated anti-MHC II (eBioscience, Hatfield, UK), APC–Cy7-conjugated anti-CD11b (BD Biosciences), APC-conjugated anti-F4/80 (eBioscience), PE-conjugated anti-CD103 (eBioscience) and biotinylated anti-CD11c antibody (BD Biosciences) followed by Qdot 605-conjugated streptavidin (Invitrogen, Paisley, UK). Cells were washed and acquired on a LSRII flow cytometer (BD Biosciences).

## Results

**Figure 1:**
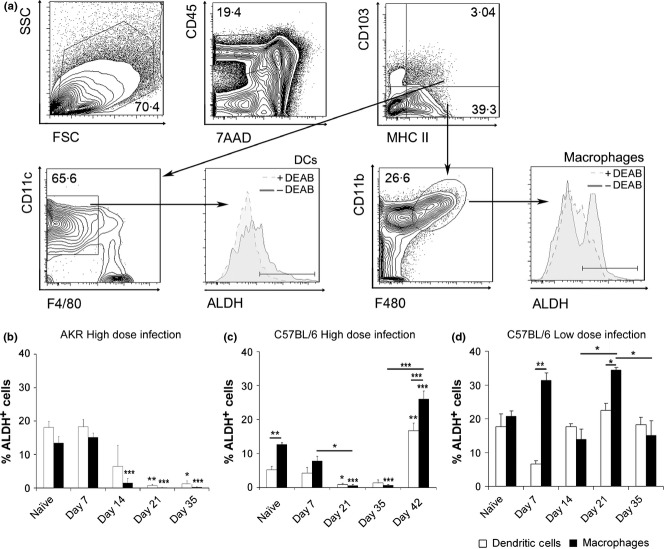
Aldehyde dehydrogenase (ALDH) activity is locally decreased during high-dose *T. muris* infection. Lamina propria leukocytes (LPLs) were isolated from the large intestines of susceptible AKR mice and resistant C57BL/6 mice at weekly intervals throughout the course of *T. muris* infection. (a) Gating strategy for 7AAD^-^CD45^+^MHCII^+^CD103^-^F4/80^+^CD11b^+^ macrophages and 7AAD^-^CD45^+^MHCII^+^CD103^+^F4/80^-^CD11c^+^ dendritic cells that were positively stained for ALDH activity. The blue line represents ALDH^+^ cells and the red line shows ALDH^+^ activity incubated with the retinaldehyde dehydrogenase inhibitor, diethylaminobenzaldehyde (DEAB), as a negative control. Percentage of MHCII^+^CD103^-^F4/80^+^CD11b^+^ macrophages or MHCII^+^CD103^+^F4/80^-^CD11c^+^ dendritic cells that have ALDH activity at each stage of infection in AKR mice given a high-dose (chronic) infection (b), C57BL/6 mice given a high-dose (acute) infection (c) and C57BL/6 mice given a low-dose (chronic) infection (d). Graphs show means + SEM. (*n* = 3–4) *P < 0·05, **P < 0·01, ***P < 0·001, compared to naïve levels, unless otherwise shown. Data is representative of two independent experiments, repeated at key time points.

To establish whether the production of retinoic acid changes over the course of an acute and/or chronic *T. muris* infection, the ALDEFLUOR assay was used to measure the expression of aldehyde dehydrogenases (ALDH) in large intestinal macrophages and dendritic cells (DCs). ALDH enzymes are responsible for the production of retinoic acid from its precursors, and their expression is tightly regulated 13. Acute *T. muris* infection was modelled in C57BL/6 mice given a high-dose infection (expulsion complete by day 21 p.i.). Chronic infection was modelled in both AKR mice given a high-dose infection and C57BL/6 mice given a low-dose infection. Thus, lamina propria leucocytes were isolated from the colons of chronically infected AKR mice, chronically infected C57BL/6 mice and acutely infected C57BL/6 mice at 7-day intervals and FACS stained. The phenotyping of gut macrophages and DCs is complex and controversial 18, 19. In this study, macrophages were defined as 7AAD^-^CD45^+^MHCII^+^CD103^-^F4/80^+^CD11b^+^ cells and DCs were defined as 7AAD^-^CD45^+^MHCII^+^CD103^+^F4/80^-^CD11c^+^ cells (Fig. 1a). Percentages of macrophage and DC populations expressing ALDH were analysed rather than total numbers of cells, as is it impossible to determine total numbers of cells given the way cells were extracted from the gut.

In AKR mice, worms were not expelled (data not shown). In these mice, the percentages of DCs that expressed ALDH enzymes diminished post-infection and were significantly reduced from naïve levels at day 21 (*P* < 0·01) and day 35 (*P* < 0·05) p.i. (Fig. 1b). Mirroring the changes seen in DCs, the percentage of macrophages that had ALDH activity decreased over the course of infection and was significantly lower than naïve levels from day 14 onwards (*P* < 0·001 at day 14, 21 and 35) (Fig. 1c). Furthermore, the percentages of DCs and macrophages possessing ALDH activity were not significantly different from each other (Fig. 1b).

In C57BL/6 mice given a high dose of *T. muris* eggs (acute infection), worms were expelled by day 21 (data not shown). In these mice, the percentage of DCs that had ALDH activity diminished over the early stages of infection and were significantly reduced from naïve levels at day 21 (*P* < 0·05) (Fig. 1c). However, at day 35 p.i., the percentage of ALDH^+^ DCs started to rise again, and at day 42 p.i. (approximately 2 weeks after worm expulsion), the percentages were significantly higher than naïve levels (*P* < 0·01) (Fig. 1c). The percentage of macrophages that had ALDH activity decreased throughout the acute infection and were significantly lower than naïve levels at day 21 and day 35 p.i. (*P* < 0·001 for both) (Fig. 1c). Similar to ALDH^+^ DCs, the percentage of macrophages expressing RALDH rose at day 42 p.i. and were also higher than those at the naïve time point (*P* < 0·01) (Fig. 1c). Moreover, unlike in chronically infected AKR mice, the percentage of macrophages that had ALDH activity were higher than the percentage of ALDH^+^ DCs in C57BL/6 mice (*P* < 0·01 in naïve mice and *P* < 0·001 at day 42 p.i.), suggesting that perhaps macrophages and not DCs are the major source of retinoic acid in C57BL/6 mice during acute *Trichuris* infection (Fig. 1c).

In C57BL/6 mice given a low dose of *T. muris* eggs to establish a chronic infection, the percentage of DCs with the potential to produce retinoic acid remained the same throughout the time course of *T. muris* infection. The percentage of ALDH^+^ macrophages increased at day 21 p.i. compared with day 14 and 35 levels (*P* < 0·05 for both); however, none of the percentages of RALDH^+^ macrophages or DCs were significantly changed from their corresponding naïve levels (Fig 1d). Collectively, these results indicate that a high level *T. muris* infection leads to a reduction in the percentage of DCs and macrophages expressing ALDH, which is restored after worm expulsion. Further, the data indicate that there may be a threshold of worm-induced inflammation required to trigger the decrease in the percentage of cells expressing ALDH.

## Discussion

In this study, we show, for the first time, that the number of macrophages and DCs expressing ALDH enzymes (the enzymes important in retinoic acid production) is significantly decreased locally in the gut during an ongoing *T. muris* infection, both in a chronic model of infection and at early stages of an acute infection. This is in line with previous work that has described a decreased RALDH2 expression and activity in CD103^+^DCs from MLNs in colitic mice compared with steady-state controls 16. Our study extends this work, however, in that we find that the reduction in ALDH^+^ gut macrophages and DCs, triggered by inflammation, is absent at later stages of an acute infection. Indeed, percentages of ALDH^+^ gut macrophages and DCs are significantly higher than naïve levels at day 42 p.i. when the parasite has been expelled and the inflammation is resolving. Recent work has shown that IL-4 can drive RALDH2 expression in alternatively activated macrophages in the liver of *Schistosoma mansoni*-infected mice 20. The alternative activation of macrophages has been shown to peak around day 21 p.i. with *T. muris* 21, as does Th2 cytokine production in resistant mouse strains 4, and so this may explain the increase in ALDH^+^ expression that we observe during the later stages (e.g. day 42 p.i.) of an acute *T. muris* infection. Interestingly, we also find that infection with a low dose of *T. muris* eggs seemed to have no effect on the potential of macrophages and DCs to produce retinoic acid, suggesting that a threshold of inflammation exists above which the production of retinoic acid is locally reduced.

Given the role played by retinoic acid in influencing the Th1/Th2 balance 10, any local reduction in available RA through a decrease in the number of cells expressing ALDH enzymes may be a regulatory mechanism employed by the parasite to prevent host protective immunity, although the potential to produce retinoic acid is also decreased in nonparasitic models of colitis 16. Alternatively, the reduction in local retinoic acid production may simply reflect a lack of absorption of vitamin A into the gut during the worm infection, given the localization and disruptive nature of the parasite. Lack of vitamin A and hence substrate may cause a down-regulation of ALDH enzymes in macrophages and DCs. Indeed, previous work has shown that ALDH enzyme expression is decreased in small intestinal lamina propria DCs during vitamin A deficiency 22. If retinoic acid is a driver, rather than a regulator of inflammation 23, it may also be possible that the reduction in locally available retinoic acid is a host protective mechanism to prevent excessive damage to host tissues.

The fine regulation of retinoic acid production seems to be an important mechanism in the control of immune responses. Reports have shown that varying concentrations of retinoic acid can alter immunity in opposing fashions. For example, high concentration of retinoic acid can block Th17 cell differentiation 24, whilst low concentrations have been shown to be essential for the differentiation of these cells 25. Thus, the changes in populations of gut leukocytes positive for the enzymes responsible for retinoic acid production that we observe during *T. muris* infection extend our current understanding of the relationship between vitamin A and intestinal parasitic infections. Indeed, this knowledge suggests that immune responses to *T. muris* may be able to be modulated through addition of exogenous vitamin A or retinoic acid during the periods of reduced endogenous production, although further investigation is needed to explore this.

In conclusion, we show, via measuring ALDH expression in macrophages and DCs, that the potential to produce retinoic acid locally decreases *in vivo* in the face of helminth-driven inflammation and that production returns to normal levels following expulsion of the parasite and resolution of inflammation. We suggest that a threshold level of inflammation is required to drive the decrease in ALDH expression, as no changes in the percentage of immune cells expressing ALDH enzymes are observed during a low-level parasite infection.
